# Large Language Model–Assisted Semiautomated Data Harmonization Pipeline for the Multiple Chronic Disease Disparities Research Consortium: Framework Development and Comparative Validation Study

**DOI:** 10.2196/93996

**Published:** 2026-10-22

**Authors:** Hyelee Kim, Shuang Liang, Kathy Lanier, Sarit Helman, Nancy F Cheng, Niloufar Ameli, Jaysón Davidson, Shivani Mehta, Yulin Hswen, Catherine E Oldenburg, Kim F Rhoads, Edwin D Charlebois, Stuart A Gansky, William Brown III

**Affiliations:** 1 Department of Epidemiology and Biostatistics, University of California, 550 16th St., Floor 2, San Francisco, CA, 94143, United States; 2 Division of Preventive Science, Department of Medicine, University of California, San Francisco, CA, United States; 3 Research Coordinating Center for Reducing Disparities in Multiple Chronic Diseases, University of California, San Francisco, CA, United States; 4 Computational Precision Health, University of California San Francisco and University of California Berkeley, Berkeley, CA, United States; 5 Social and Behavioral Sciences Branch, Division of Intramural Research, Eunice Kennedy Shriver National Institute of Child Health and Human Development, BG 6710 Rockledge Dr Wing B, Bethesda, MD, 20817, United States, 1 301-555-6677; 6 Division of Oral Epidemiology & Dental Public Health, University of California, San Francisco, CA, United States; 7 Pharmaceutical Science and Pharmacogenomics program, University of California, San Francisco, CA, United States; 8 Bakar Computational Health Sciences Institute, University of California, San Francisco, CA, United States; 9 College of Computer Mathematical and Natural Science, Department of Epidemiology and Biostatistics, School of Public Health, The Artificial Intelligence Interdisciplinary Institute at Maryland (AIM), University System of Maryland, College Park, MD, United States; 10 Francis I. Proctor Foundation, University of California, San Francisco, CA, United States; 11 Massey Comprehensive Cancer Center, Virginia Commonwealth University, Richmond, VA, United States

**Keywords:** large language model, human-in-the-loop, semiautomation, data harmonization, common data element, social determinants of health

## Abstract

**Background:**

The increasing availability of machine-readable research data has created a growing need for efficient large-scale data harmonization (DH). Although large language models (LLMs) show promise for reducing the time and labor required for DH, their effective integration into harmonization workflows remains an important methodological challenge.

**Objective:**

This study aims to develop and evaluate a semiautomated, human-in-the-loop (HITL) LLM-assisted DH workflow as a proof of concept across 8 projects within a research consortium focused on disparities in multiple chronic conditions.

**Methods:**

We developed an LLM-assisted DH workflow that combined preprocessing, semantic mapping, variable response mapping, synthetic data generation, data transformation, and iterative researcher review. Using Generative Pretrained Transformer (GPT-4o) hosted in a secure academic environment, project-specific survey items were mapped to 114 Consortium Common Data Element (CDE) semantic groups. Semantic mapping accuracy was evaluated based on the Consortium’s consensus harmonization results. Complete and incomplete semantic mappings were distinguished according to construct overlap, and incomplete mappings requiring context-dependent human judgment were excluded for mapped item pairs and CDE semantic groups with and without HITL review.

**Results:**

Following preprocessing, 884 survey items were included in the DH workflow, of which 812 were mapped to a mean of 79 CDE semantic groups per project. Mean semantic mapping accuracy for item pairs was 95.9% (SD 5.7%) with HITL review and 86.7% (SD 8.6%) without HITL review. At the CDE semantic-group level, the corresponding accuracies were 98.9% (SD 1.7%) and 94.9% (SD 4.0%), respectively. Across projects, omission of valid semantic mappings occurred more frequently than incorrect semantic mappings, particularly for heterogeneous response structures and subjective constructs measured using different instruments. Pipeline components—including concept-based preprocessing, iterative prompt refinement, and targeted HITL review—improved mapping completeness and supported variable harmonization across heterogeneous datasets.

**Conclusions:**

This proof-of-concept study demonstrates that the effectiveness of LLM-assisted DH depends more on the design of a structured HITL workflow rather than on the LLM alone. Combining preprocessing, iterative verification, and targeted human oversight improved the efficiency and reliability of DH while preserving researcher judgment for complex mapping decisions.

## Introduction

### Background

Data harmonization (DH) enables the integration of individual-level data across studies, increasing statistical power to address complex biomedical and public health research questions [1,2]. Unlike approaches like meta-analysis [3] or federated learning [4,5], which combine study-level results or trained models, DH directly integrates participant-level data for a wide range of research and clinical applications. Over the past 2 decades, numerous approaches have been developed to improve DH across research domains [2,6-10]. These include standardized information technology platforms and Common Data Models (CDMs), such as BioSHaRE (Biobank Standardization and Harmonization for Research Excellence in the European Union), the Observational Medical Outcomes Partnership (OMOP) CDM in the All of Us, and National Patient-Centered Clinical Research Network (PCORnet) by the Patient-Centered Outcomes Research Institute (PCORI) [7,11-16].

Despite these advances, semantic heterogeneity remains a major challenge because similar constructs are often collected using different question wording, response options, or levels of granularity [9]. As a result, harmonization frequently requires subjective human judgment and may result in incomplete or inconsistent mappings. To improve data consistency and interoperability, standardized data content and formats should ideally be established before data collection [10,17]. Reflecting this need, the National Institutes of Health (NIH) introduced the 2023 NIH Data Management and Sharing Policy, which recommends the use of common data elements (CDEs) to promote standardized, interoperable, and reusable data in NIH-funded research [18].

The Multiple Chronic Disease Disparities Research (MCD-DR) Consortium (hereafter referred to as the Consortium), also known as the Health Excellence Action Network, is an initiative funded by the National Institute on Minority Health and Health Disparities (NIMHD) [19]. Its mission is to prevent and manage multiple chronic conditions among populations disproportionately affected by health disparities [20,21]. The Consortium comprises 11 regional research centers funded through P50 Specialized Center grants, community partners, and the University of California San Francisco (UCSF) Research Coordinating Center, funded through a U24 Resource-Related Project cooperative agreement. To support data integration across diverse studies, the Consortium developed a bidirectional DH framework that combines retrospective harmonization for projects initiated before Consortium CDEs were established with prospective harmonization for projects launched afterward [19]. Unlike previous large research consortia, which have primarily relied on retrospective harmonization after project-specific data collection was completed [2,9], this framework enables harmonization throughout the lifecycle of an ongoing research consortium.

Successful implementation of the Consortium’s bidirectional DH framework required sustained collaboration between the Research Coordinating Center and community research centers to build trust and support community-based participatory research and establish consensus on CDEs [19]. To facilitate this process, the Research Coordinating Center established the CDE and DH Working Group (hereafter, the Working Group), which included investigators from the Research Coordinating Center and the P50 Centers. Through an iterative consensus process, the Working Group developed a recommended CDE set comprising 55 concepts (43 required and 12 high-value optional) across multiple domains of social determinants of health (also referred to as social drivers of health; SDOH) based on the NIMHD Research Framework [22]. The SDOH domains encompassed demographics, neighborhood and physical environment, food insecurity, social and community context, education attainment, economic security, discrimination, disability, and health and clinical care [23]. These were operationalized into 178 questionnaire items from the existing validated toolkits (eg, Phenotypes and eXposures [PhenX] Toolkit and Patient-Reported Outcomes Measurement Information System [PROMIS] Global Health, the Accountable Health Communities Health-Related Social Needs Screening Tool, and the All of Us Research Program SDOH Module) (Table S1 in Multimedia Appendix 1). Details of the CDE data dictionary are publicly available on the Consortium website-. For prospective harmonization, participating projects were strongly encouraged to incorporate these CDEs into their project-specific data dictionaries to improve interoperability and facilitate data reuse.

Despite these efforts, DH remains labor-intensive and time-consuming because harmonizing semantically heterogeneous variables requires both domain expertise and iterative consensus building [24]. The process also demands substantial coordination among researchers and community partners [24]. Consequently, developing approaches that reduce the time and resource burden of manual CDE mapping while maintaining mapping accuracy and supporting the internal validity of harmonized data has become an important priority.

### Related Work

Information on SDOH is often embedded within unstructured sources, such as clinical notes or datasets collected for purposes other than SDOH research [25]. Consequently, natural language processing (NLP) and large language models (LLMs) have demonstrated strong performance in extracting SDOH information from electronic health records (EHRs). For example, 1 study [26] reported that LLMs captured 94% of SDOH information (eg, employment, housing, and social support), whereas the *ICD-10* (*International Statistical Classification of Diseases, Tenth Revision*) codes captured only 2% based on human annotation. These findings suggest that LLMs can effectively identify semantically related concepts despite heterogeneous data representations.

Building on these capabilities, researchers have explored NLP-based approaches to reduce the manual effort required for DH. Traditional NLP methods use rule-based techniques, such as exact and fuzzy matching, together with semantic text representations (ie, embeddings). For instance, Urbanowicz et al [27] applied exact and fuzzy matching to harmonize 37,105 medical history records and 58,170 adverse event records from 21 randomized controlled trials of pulmonary arterial hypertension and chronic thromboembolic pulmonary hypertension. Automated exact matching replaced manual mapping for 74.9% of medical history records and 85.5% of adverse event records, substantially reducing manual annotation. Similarly, Blom et al [28] used a skip-gram model pretrained on English-language Wikipedia to generate semantic embeddings from metadata, including item names, definitions, response options, to harmonize patient-reported outcome measures across datasets of patients with multimorbidity.

More recently, transformer-based models have been adopted for large-scale DH tasks [29-31]. For example, Zhou et al [30] used BERT (Bidirectional Encoder Representations from Transformers) and a knowledge graph to harmonize cross-institutional EHRs by aligning heterogeneous coding systems across 2 health care institutions. Their approach achieved an area under the receiver operating characteristic curve (AUROC) of 0.92 for identifying similar entity pairs. Mapping performance varied by data type, with medication codes achieving 91.0%‐97.5% accuracy and laboratory codes achieving 59.1%‐75.8% accuracy.

Additionally, researchers have begun applying LLMs not only to information extraction but also broader DH tasks to improve scalability and efficiency [32-34]. For instance, Cho et al [32] reported that a fine-tuned small language model (eg, Mistral 7B) outperformed larger LLMs in extracting cancer staging information. In another study, researchers at the National Institute on Aging also used Generative Pretrained Transformer (GPT-4) in the AI-assisted Data Inventory and Verification Environment for Research (DIVER) platform (Center for Alzheimer’s and Related Dementias, NIH, and Data Tecnica LLC) to develop CDEs from multiple sources, including existing studies, ontologies, and medical coding systems [33]. The researchers used LLMs to generate CDEs from 31 datasets and reported that 94% of the generated metadata fields required no revision.

In a preliminary study [35], we compared the 2 LLM-based pipelines for semantic mapping of data dictionaries from 5 Consortium projects: 1) the zero-shot OpenAI GPT-4-turbo model accessed through an API, and 2) the chat-based version of the same model. The chat interface supports interactive natural-language communication, whereas the API enables fully automated workflows through programming languages, such as Python (Python Software Foundation) or R (R Foundation for Statistical Computing). For identifying the best-matched CDE items for 1305 project questionnaire items, the chat-based approach required approximately 5 days, whereas the fully automated API pipeline completed the task in just 1 day (excluding time required to learn the LLM and develop the Python scripts). The API-based approach achieved a mean semantic mapping accuracy of 80.3% (SD 10.0%) at an additional API cost of US $87 for LLM, while the free chat-based version achieved a slightly higher mean accuracy of 86.6% (SD 13.0%). Performance varied according to the characteristics of each project’s data dictionary, suggesting that no single LLM workflow was optimal for all harmonization tasks. Furthermore, the large volume of metadata made it difficult to identify LLM-generated omissions during downstream harmonization. These findings suggested that a semiautomated, human-in-the-loop (HITL) workflow could better balance efficiency, accuracy, and completeness than either approach alone.

### Study Objectives

During implementation of the Consortium’s bidirectional DH framework, advances in LLMs presented an opportunity to reduce the labor-intensive process of mapping heterogeneous study variables to Consortium CDEs (Table 1). Rather than evaluating the LLM in isolation, this proof-of-concept study evaluated the feasibility of integrating LLMs into a structured HITL DH workflow that combines preprocessing, semantic mapping, variable response mapping, and iterative researcher review. Specifically, we examined whether LLM-assisted harmonization could improve the efficiency and scalability of semantic mapping while maintaining expert oversight and mapping accuracy.

**Table 1.** Lists and examples of heterogeneities in data dictionaries.

Questionnaire example A	Questionnaire example B
List 1. Question for the responder or the family, including the responder (response for families and responder could be different)	
In the last 12 months, did you or other adults in your household ever cut the size of your meals or skip meals because there wasn’t enough money for food?	In the last 12 months, did you ever cut the size of your meals or skip meals because there wasn’t enough money for food?
List 2. Differences in branching logic (changes in meaning by changing the denominator of the variable)	
How often did this happen - almost every month, some months but not every month, or in only 1 or 2 months? A question was asked in the condition:[12_mos_change_diet] =“1”	The same food insecurity question was asked in these conditions: [12_mos_food_money_freq]=“1” or [12_mos_food_money_freq]=“2”or [12_mos_afford_balanced_meals]=“1” or [12_mos_afford_balanced_meals]=“2” or [12_mos_change_diet]=“1”
List 3. Differences in response options (we need household size up until 20 to identify the federal poverty levels)	
Household size: 1, 2, 3, 4, 5, 6 or more	Household size: response options up until 20
List 4. Differences in response options and duration asked in the question (we need to make a consensus if we are going to harmonize these variables and how to map them)	
Over the past 7 days, how often did you engage in moderate sport and recreational activities such as doubles tennis, ballroom dancing, hunting, ice skating, golf without a cart, softball or other similar activities? 0: Never 1: Seldom (1‐2 days) 2: Sometimes (3‐4 days) 3: Often (5‐7 days)	In the last 30 days, other than the activities you did for work, on average, how many days per week did you engage in moderate exercise (like walking fast, running, jogging, dancing, swimming, biking, or other similar activities)? 0, 0 1, 1 2, 2 3, 3 4, 4 5, 5 6, 6 7, 7
List 5. Asking in a single question or multiple questions (If there are projects that used a combined questionnaire, we cannot harmonize the questions separately)	
Has lack of transportation kept you from medical appointments or from getting medications? Has lack of transportation kept you from doing things needed for daily living? Has lack of transportation been a problem for you?	In the past 12 months, has lack of reliable transportation kept you from medical appointments, meetings, work or from getting things needed for daily living?
List 6. Asking about depression using different questionnaires (first question asks about anxiety or depression, whereas the second question asks symptoms of depression)	
Anxiety or depression I am not anxious or depressed I am slightly anxious or depressed I am moderately anxious or depressed I am severely anxious or depressed I am extremely anxious or depressed −99, skip	Over the past 2 weeks, how often have you been bothered by any of the following? Feeling down, depressed, or hopeless? Not at all Several Days More than half the days Nearly every day
List 7. Asking about stress using different questionnaires (we need to decide whether and how to map these questions)	
Questions from the PSS^a^ In the last month, how often have you felt that you were unable to control the important things in your life? In the last month, how often have you felt confident about your ability to handle your personal problems? In the last month, how often have you felt that things were going your way? In the last month, how often have you felt difficulties were piling up so high that you could not overcome them?	Question from the Accountable Health Communities Health-Related Social Needs Screening Tool Stress means a situation in which a person feels tense, restless, nervous, or anxious, or is unable to sleep at night because his or her mind is troubled all the time. Do you feel this kind of stress these days?
List 8. Differences in response option values (response mapping needs additional attention to avoid incorrect value mappings)	
Birth state 1, Alabama 2, Alaska 3, Arizona 4, Arkansas 5, California 6, Colorado 7, Connecticut 8, Delaware 9, District of Columbia 10, Florida **:** 50, Wisconsin 51, Wyoming	Birth state 1, Alabama 2, Alaska 3, Arizona 4, Arkansas 5, California 6, Colorado 7, Connecticut 8, Delaware 9, Florida **:** 49, Wisconsin 50, Wyoming 51, Washington, D.C.
List 9. Differences in question structures (one of the examples that may be missed by human researcher)	
Does it limit your activities? (Rheumatoid arthritis, 1: Yes, 0: No)	Do you have chronic muscular or joint issues that cause pain and physical limitation? 1, Yes 0, No 2, Don’t know/ Unsure If yes, what is causing the pain? (Mark all that apply) Lupus (systemic lupus erythematosus) Polymyalgia rheumatica Osteoarthritis Rheumatoid Arthritis Other, specify: {muscular_issue_oth} Don ’ t know/ Unsure

a PSS: Perceived Stress Scale.

## Methods

### Data: Consortium CDE and Project Dictionaries

The Consortium CDE dictionary contains 178 items, of which 154 were eligible for mapping. The eligible CDE items consisted exclusively of survey questionnaire items and did not include vital signs, laboratory results, or biospecimen data. Twenty-four items, such as record identifiers and questionnaire instructions, were excluded.

To facilitate semantic mapping, the study authors (HK and SAG) manually grouped the 154 eligible CDE items into 114 discrete CDE semantic groups according to their broader CDE concepts. These semantic groups were developed specifically for this study to provide an appropriate level of granularity for evaluating semantic mapping accuracy and were not part of the Consortium’s original CDE harmonization process. The resulting semantic groups comprised 99 required groups and 15 high-value-optional groups and served as the reference standard for evaluating LLM semantic mapping performance (Table S1 in Multimedia Appendix 1). Although the Consortium Working Group developed a standardized CDE dictionary, the number and types of CDE semantic groups represented in each project varied depending on investigator decisions and the timing of project initiation (ie, before or after finalization of the Consortium CDE dictionary).

Among the 41 Consortium projects eligible for CDE mapping (42 projects at the time of writing [July 2026]), we selected 8 studies, each representing a different regional research center (P50), for this proof-of-concept evaluation [19]. The characteristics of the included studies are summarized in Table S2 in Multimedia Appendix 2. Because 7 of the 8 participating projects were initiated before the Consortium CDE dictionary was finalized, most projects had already developed study-specific data dictionaries. Consequently, substantial variation in questionnaire content and metadata existed across projects, necessitating semantic DH.

### LLM-Assisted DH Pipeline With HITL

The DH pipeline consisted of 4 major steps [1] semantic mapping [2], variable response mapping [3], applying the mapping to synthetic project data, and [4] pooling mapped variables and restructuring existing CDEs (Figure 1). Semantic mapping identified project-specific questionnaire items representing the same or compatible constructs as Consortium CDEs. Variable response mapping determined how project-specific response categories should be transformed to align with standardized CDE response formats. The pooling and restructuring step ensured that harmonized variables comprehensively captured each CDE while preserving compatibility with heterogeneous project data structures.

**Figure 1.**
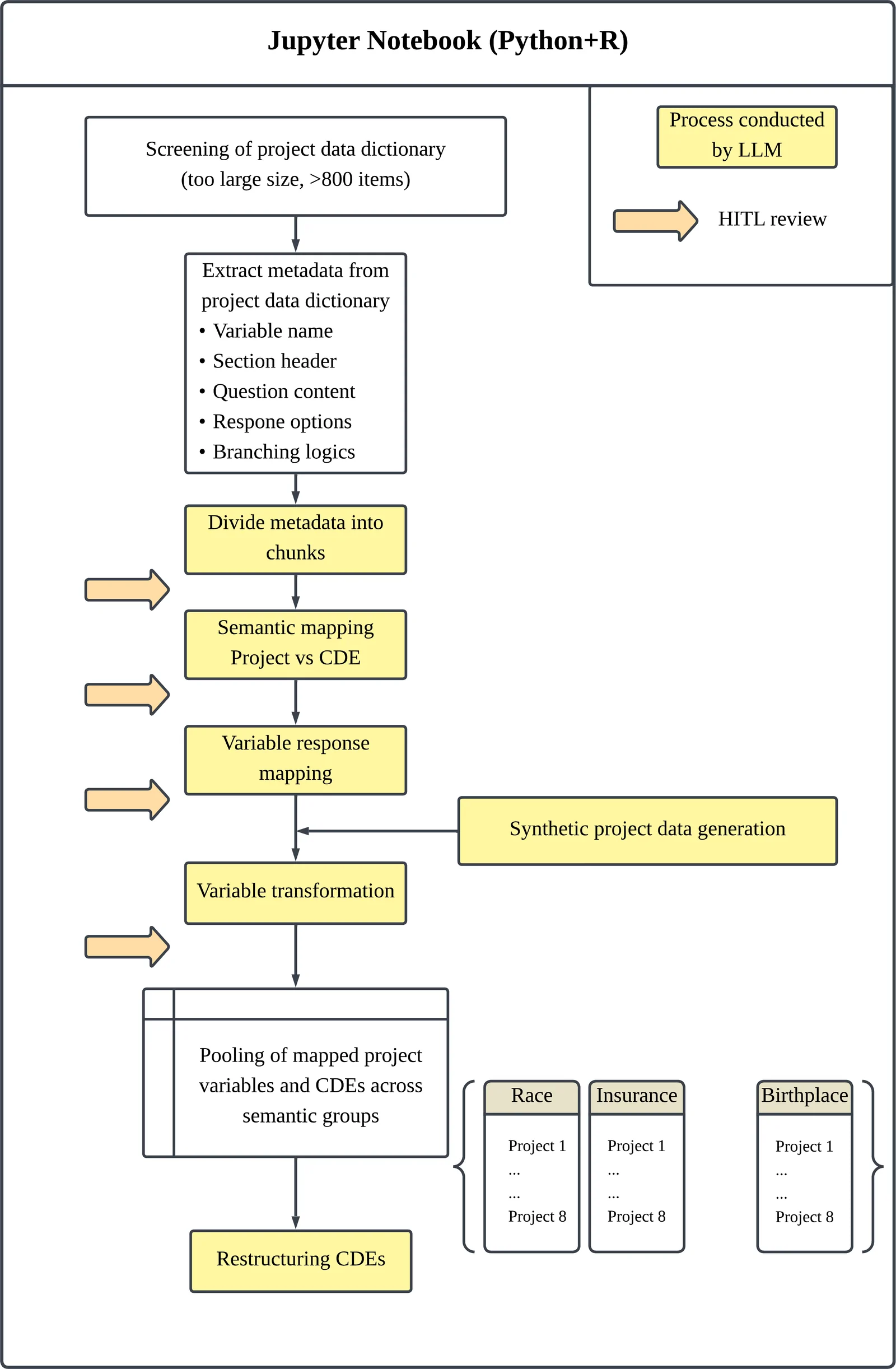
Flowchart of LLM-assisted CDE harmonization workflow. Python was used for preprocessing data dictionaries, including NLP tasks and interactions with the OpenAI API, whereas R was used for data wrangling and transforming synthetic project data into the Consortium CDE formats. CDE: common data element; HITL: human-in-the-loop; LLM: large language model; NIL: natural language processing.

We implemented a semiautomated, HITL workflow that was adapted to characteristics of each project’s data dictionary, including its size, structure, similarity to the Consortium CDE dictionary, and results from previous steps. Rather than applying a fully automated workflow across all projects, we iteratively refined the pipeline, prompts, and programming code to improve mapping accuracy and minimize errors. The chat-based LLM interface was used to prototype prompts, simulate outputs, and assist with code development for the API-based workflow. Researcher oversight was incorporated throughout the pipeline by embedding verification steps into prompts (eg, instructing the LLM to report the number of processed items to detect potential omissions).

In the pipeline we used the OpenAI GPT-4o model through the UCSF Versa platform (University of California) [36], a secure Microsoft Azure AI environment that supports the Health Insurance Portability and Accountability Act (HIPAA)-compliant processing of sensitive research data (37). Although no personally identifiable information was included in this study, project data dictionaries were harmonized within the secure Versa environment because they were not publicly available. In addition, interactions conducted through the UCSF Versa platform were not retained by the hosted models, thereby protecting data security and privacy. To support reproducibility and collaboration, the API-based workflow was implemented in Jupyter Notebook (version 7.2.2; Project Jupyter) [38], which provides an interactive computational environment for Python and R. Python (version 3.9.21) was used for preprocessing data dictionaries, including NLP tasks and interactions with the OpenAI API, whereas R (version 4.4.2) was used for data wrangling (eg, using the *dplyr* package) and transforming synthetic project data into the Consortium CDE formats [39,40]. This combined workflow leveraged the complementary strengths of both programming languages.

### Data Preprocessing

Preprocessing procedures were adapted to the size and structure of each project’s data dictionary. For dictionaries containing more than 800 questionnaire items, researchers first performed a broad manual screening to identify relevant sections. Metadata from the projects’ data dictionaries—including variable names, section headers, question content, response options, and associated branching logic—were programmatically extracted from the CSV-formatted data dictionaries using Python. Based on findings from our preliminary study [35], the extracted metadata were divided into concept-based, manageable chunks before semantic mapping to accommodate the token size limitations of LLMs. For example, because comorbidity items represented the largest CDE concept (42 of 154 CDE items), the LLM first identified comorbidity-related items (Textbox 1). Additional chunks were created for demographic variables (eg, race, ethnicity, sex, and age) and the remaining questionnaire items.

Textbox 1. **Table of example prompts used in the pipeline.**

**Task**

You are a clinical informaticist who will conduct semantic mapping of 2 datasets.

Identifying comorbidity items in the data dictionary

Below is a data dictionary.

Provide a list_A of comma-separated, quoted ‘variable_name’ for comorbidities, treatment, and functional limitations, including total numbers.

Provide a list_B of comma-separated, quoted ‘variable_name’,‘variable_name,’ which are not for comorbidities, treatment, and functional limitations, including total numbers.

Provide a list_C which is not included in list_A or list_B.

List A: ’scq_covid19’,‘scq_covid19,’’scq_heart'’

**Explanation**

- Although this prompt was aimed to identify item lists for comorbidities and the related questions (treatment and functional limitations), we want to see the rest of the items to see if there were any omitted items in the list A
- Additionally, we wanted only variable names included in the list. Therefore, we provided the example list to the model
- To prevent unexpected omissions, we requested to provide the numbers in each list

**Task**

Identifying the variable response mapping

'df_vt'’ is the data dictionary for matched variables from CDE and project.

Included information: variable name, section header, question content, response, branching logic

'project_b'’ is a project data (includes missing observation). We have a csv file of project_b. No need to include code to generate '’project_b'’

Provide an R code to transform a project variable presented in former columns to the CDE variable presented in latter columns in the same row. Name it '’transformed_data'’

The beginning of R code includes copying ‘project_b’ data frame as '’transformed_data'’

**Explanation**

- We informed which information each data dictionary contains.
- We clarified that we do not need coding for generating synthetic data to prevent unnecessary token usage.
- We provided information about the position of CDE and project presented in a same row
- To maintain consistency in coding, we instructed to start from copying the project data.

During the previous study, we observed that instructional text was occasionally mapped to questionnaire items because of semantic similarity. For example, instructions preceding family income questions were incorrectly mapped to the family income question CDE. To address this issue, we restructured the metadata by incorporating relevant instructions into the corresponding section headers rather than treating them as separate items, enabling the LLM to better capture the relationships between instructions and questionnaire items. The semantic harmonization analysis was limited to English-language questionnaire content. When project data dictionaries contained bilingual (English and Spanish) questionnaire items, only the English text was included in the LLM input to reduce token length and improve processing efficiency. The token size for CDE dictionary was divided into 3 chunks: comorbidities (2.4 thousand tokens), demographics (3.7 thousand tokens), and all remaining items (10.9 thousand tokens), resulting in a total of 17.0 thousand tokens.

### Semantic Mapping Using LLMs

For semantic mapping, metadata from both the Consortium CDE dictionary and each project data dictionary, including variable names, section headers, questionnaire items, response options, and branching logic, were provided to the LLM as structured prompt input organized into concept-based chunks (eg, comorbidities, demographics, and remaining questionnaire items) (Figure 1). The LLM was then prompted to identify semantically corresponding questionnaire items and generate mapping clusters between the project and Consortium CDE dictionaries.

Several strategies were additionally incorporated to improve mapping performance. Prompts required the LLMs to explicitly report unmapped items to minimize omissions, and additional instructions clarified item ordering and formatting to reduce ambiguity. Following each mapping task, researchers reviewed the LLM-generated clusters, corrected mapping errors if necessary, and approved the results before proceeding to variable response mapping. Mapped items were organized according to CDE item numbers, whereas unmapped project items were flagged for further review to determine whether additional harmonization was possible. This process was repeated for every chunk across all 8 projects.

For evaluation, semantic mapping results were classified as either complete or incomplete according to the degree of conceptual overlap between project items and the corresponding CDEs (ie, complete vs partial construct overlap).

### Variable Response Mapping, Data Transformation, and Harmonized Variable Restructuring

Semantic mapping results were stored in 2 complementary formats: [1] CDE semantic group-specific files (one file for each of the 114 semantic groups), and [2] project-specific mapping files (one file per project). Project-specific mapping files were initially used to transform project variables into the corresponding Consortium CDE formats. However, differences in response categories across projects often required additional harmonization. For example, the Consortium CDE for educational attainment included more detailed response categories than those collected in some individual projects. To establish consistent harmonization rules, project variables belonging to the same CDE semantic group were consolidated across projects and reviewed collectively. The LLM then analyzed the grouped variables, identified similarities and differences in response structures, and suggested candidate harmonization strategies while highlighting discrepancies among projects (Textbox 2).

Textbox 2. **Example of items related to a discrete Common Data Element (CDE) construct collected across the studies and large language model (LLM)’s analysis for data harmonization (DH).**

Prompt

'df'’ is the data dictionary for matched variables from CDE and projects.

Included information: variable name, section header, question content, response, branching logic

Based on the information, could you suggest how to harmonize the presented variables? Develop a new CDE item that can harmonize them.

If you find any problems from harmonizing them, describe what they are.

Response

To harmonize the presented variables from different projects, we need to create a comprehensive set of questions and responses that can incorporate the different aspects covered by these datasets. We can start by assessing the commonalities and differences in the datasets and then suggest a unified approach.

### Commonalities:

**Domain**: Most datasets consider trouble paying for basic needs such as housing, heat and electricity, medical needs, transportation, childcare, and debts.
**Response Options**: The response options are fairly consistent across datasets, usually including "Yes",“Yes,” "No",“No,” and a "“Prefer not to answer"” option.
**Time Frame**: Most instruments refer to the past three months.

(omitted)

### Problems and Considerations:

**Response Recoding**: The harmonized dataset will need to reconcile differences in response coding to ensure consistency, eg, standardizing "Prefer not to answer" to -88.
**Data Loss or Detail Reduction**: There is a risk of losing specific nuances provided by more detailed response options or specialized questions in the original datasets.
**Aggregation of "“Other"” Responses**: The utility of an "“Other"” category depends on the detail provided by respondents. Therefore, aggregation and harmonization may lead to merged data that lacks clarity.
**Compatibility with Existing Systems**: Harmonizing may require changes in how data systems currently store and process responses, potentially requiring reevaluation of data models.

Establishing this new harmonized CDE item will create a more comprehensive yet standardized approach, allowing for consistent cross-dataset analysis while minimizing information loss.

For variable response mapping, we used the *rpy2* package within the IPython kernel to enable seamless integration of Python and R in a single Jupyter Notebook environment. Synthetic project data (10 observations per project) were generated by the LLM from each project’s data dictionary to simulate the variable transformation procedure without actual project data. Using the semantic mapping results, the LLM generated code to transform project-specific variables into standardized Consortium CDE formats. To improve coding accuracy, transformations were generated and validated iteratively in small batches of 5‐10 variables. When errors were identified, prompts and generated code were refined before the transformation was repeated. For complex mappings involving one-to-many or many-to-many relationships (eg, a single diabetes variable containing both diagnosis and treatment information), all relevant variables were presented simultaneously to enable LLM to generate appropriate transformation rules.

Variable response mapping results were classified as either complete or incomplete. Incomplete mappings represented situations in which multiple harmonization strategies were reasonable because of differences in variable structure or response categories across studies (eg, mapping multiple-choice and single-choice race variables). These mappings were excluded from automated variable transformation in this proof-of-concept study. Throughout the pipeline, prompts and input data were iteratively refined to improve transformation accuracy. Overall, the pipeline was designed to support iterative collaboration between researchers and the LLM rather than fully automate the DH.

### Evaluation of LLM Performance

Because incomplete semantic mappings required context-dependent human judgment, they were excluded from accuracy evaluation. Such mappings often involve multiple reasonable harmonization strategies depending on the intended research question, such as harmonizing variables with different categorizations (eg, race, insurance, or comorbidities) or determining whether conceptually related but nonidentical questionnaire items should be combined. Including incomplete mappings would therefore make the evaluation dependent on subjective scoring decisions rather than objective semantic agreement. Accordingly, incomplete mappings were excluded from both the numerator and denominator when calculating semantic mapping accuracy.

To evaluate the contribution of human oversight, mapping accuracy was compared before and after researcher review (ie, with HITL correction). Semantic mapping accuracy was evaluated at 2 levels. First, accuracy was calculated for individual semantically mapped item pairs as the percentage of correctly mapped project-CDE pairs among all complete semantic mappings. Because a single project item could correspond to multiple CDE items (or vice versa), the total number of mapped pairs varied across projects depending on the final harmonization decisions.


(1) $$\begin{array}{r} \text{Accuracy of semantically mapped item pairs}\\ =\frac{\text{The number of correctly mapped pairs}}{\text{Total number of complete semantic mappings}}\times 100 \end{array}$$

Second, accuracy was evaluated at the CDE semantic-group level to account for differences in the importance and granularity of individual questionnaire items. Semantic-group accuracy was defined as the percentage of correctly mapped CDE semantic groups among all complete semantic groups represented in a project’s data dictionary (maximum of 114 groups). A semantic group was considered correctly mapped when the project’s key concepts were accurately aligned with the corresponding Consortium CDE semantic group. The 2 metrics provide complementary evaluations: item-level accuracy reflects the correctness of individual semantic mappings, whereas semantic-group accuracy assesses whether the intended CDE constructs were successfully identified.


(2) $$\begin{array}{r} \text{Accuracy of CDE semantic group}\\ =\frac{\text{The number of correctly mapped CDE groups}}{\text{Total number of complete CDE group mappings}}\times 100 \end{array}$$

The reference standard for evaluating mapping accuracy was the final semantic mapping established through the Consortium’s DH process. Because harmonization began at different times across projects, the initial mapping approach varied. One project initially used an NLP-based fuzzy matching approach, whereas another project completed semantic and variable mapping independently and shared the results with the Research Coordinating Center. Regardless of the initial approach, all projects subsequently followed the same consensus workflow. First, the initial semantic mappings generated by the LLM (GPT-4-turbo in the UCSF Versa platform) were reviewed by the individual project team to identify inaccuracies and provide domain-specific feedback. Second, the Research Coordinating Center integrated project feedback and reached internal consensus. Finally, mappings that remained controversial after this review were discussed during the Consortium’s monthly Working Group meetings. The resulting consensus mappings served as the reference standard for evaluating LLM performance. We also recorded the total API cost and processing time required to complete the harmonization workflow across all eight projects. Although prompt length was reduced when necessary (eg, by excluding duplicate Spanish-language questionnaire text) to remain within the LLM context window, token usage itself was not evaluated as a study outcome.

### Ethical Considerations

No personally identifiable information, or participant interactions were included in this study. Project metadata (ie, data dictionaries) were analyzed within the secure UCSF Versa environment because these materials were not publicly available. Access was restricted to authorized UCSF researchers. The UCSF Institutional Review Board determined that this study was exempt from review (protocol #21‐35033).

## Results

### Overall DH Performance

Across all 8 projects, the workflow successfully integrated LLM-assisted semantic mapping, variable response mapping, and iterative researcher review. Following preprocessing, 884 questionnaire items from the 8 project data dictionaries were included in the DH pipeline. Of these, 812 items (812/884, 91.9% of eligible questionnaire items and 812/903, 90.0% of all data dictionary items) were semantically mapped to a mean of 79 CDE semantic groups per project, including incomplete semantic mappings (Table S3 Multimedia Appendix 3). The number of projects represented within each CDE semantic group varied across the Consortium. Figure 2 summarizes the distribution of topic entities across projects.

**Figure 2.**
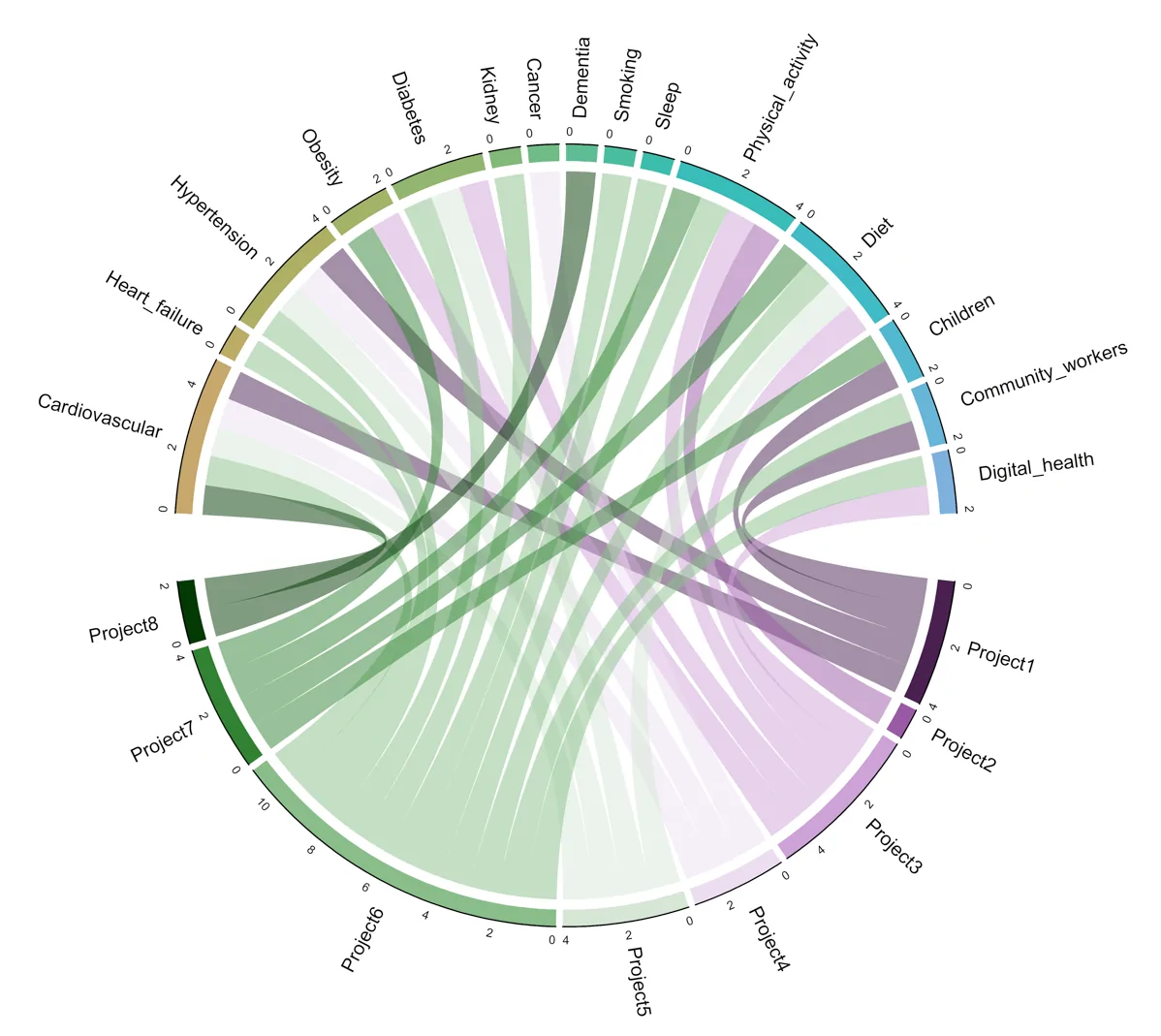
Chord diagram of topic entities included in the projects the chord diagram illustrates the relationships between the 8 consortium projects and the 15 topic entities represented in their data dictionaries. Each ribbon indicates that a project includes data related to the corresponding topic entity. For example, project 1 includes 4 topic entities: cardiovascular disease, hypertension, children, and community health workers. Likewise, the cardiovascular disease topic is represented in 5 projects (projects 1, 4, 5, 6, and 8). The diagram provides an overview of the distribution of research topics across projects and highlights similarities and differences in their thematic focus.

After excluding incomplete semantic mappings (Table S4 in Multimedia Appendix 4), the mean accuracy of semantically mapped item pairs was 95.9% (SD 5.7%) with HITL review and 86.7% (SD 8.6%) without HITL review. At the CDE semantic-group level, the corresponding mean accuracies were 98.9% (SD 1.7%) and 94.9% (SD 4.0%), respectively (Table 2). The complete LLM-assisted DH workflow across all 8 projects processed approximately 2.4 million tokens and incurred a total API cost of US $11.44. The LLM also generated recommendations for harmonizing variables with heterogeneous response structures across projects, providing guidance for variable response mapping and data transformation (Table S5 in Multimedia Appendix 5).

**Table 2.** Accuracy of semantically mapped item pairs with and without HITL^a^ according to projects^b^.

Number of eligible items	Project	Mapped item pairs (% of incomplete mappings)^c^	Item pair accuracy (%)^c^	Semantic group accuracy (%)^c^	Mapped item pairs (% of incomplete mappings)^d^	Item pair accuracy (%)^d^	Semantic group accuracy (%)^d^	Total number of mapped semantic groups
1	127	125 (0)	96.1	96.4	124 (0)	100.0	100.0	84
2	118	94 (13.8)	84.4	93.8	97 (14.4)	85.6	95.5	75
3	79	50 (12.0)	69.8	86.0	70 (11.4)	100.0	100.0	52
4	131	123 (4.9)	97.4	96.7	117 (2.6)	100.0	100.0	91
5	126	123 (0)	82.9	97.8	123 (0)	100.0	100.0	90
6	71	50 (8.0)	86.3	97.4	60 (16.7)	94.3	97.6	45
7	129	122 (0)	89.1	98.0	122 (0)	89.1	98.0	101
8	122	102 (1.0)	87.3	93.0	112 (4.5)	98.2	100.0	86

a HITL: human-in-the-loop.

b Accuracy was calculated after excluding incomplete semantic mappings. The Consortium common data element (CDE) dictionary contains 154 questionnaire items eligible for semantic mapping, grouped into 114 discrete common data element (CDE) semantic groups (99 required and 15 high-value-optional). Because projects initiated before the development of the Consortium common data element (CDE) dictionary could not modify their existing data dictionaries (ie, retrospective harmonization), the number of common data element (CDE) semantic groups represented varied across projects. Therefore, differences in the total number of mapped semantic groups shown in the final column are expected.

c Without HITL.

d With HITL.

### Project-Specific Mapping Characteristics

Although semantic mapping performance varied across projects because of differences in data dictionary content and structure, several consistent patterns emerged. Multimedia Appendix 3 represents the DH results across eight projects according to semantic CDE groups. Across all 8 projects, omission of valid semantic mapping was a more frequent source of error than incorrect semantic mappings. Omission errors occurred primarily when variables required one-to-many or many-to-many mappings or when conceptually similar constructs were measured using different questionnaires. These findings highlight the importance of researcher review during semantic mapping, particularly for heterogeneous response structures and subjective constructs.

#### Project 1

Project 1 primarily consisted of questionnaire items corresponding to the required Consortium CDEs. Minor differences included additional response options (eg, Middle Eastern and North African [MENA] race) and social needs questions introduced after the project was initiated. With HITL review, all 124 eligible items were correctly mapped to 84 CDE semantic groups without omissions. Without HITL, omission of 2 standard response-option items (ie, −77 for “Don’t know”, and −88 for “Prefer not to answer”) and incorrect mapping of COVID-19 comorbidity questions (ie, past vs current) reduced semantic group accuracy from 100% to 96.4%.

#### Projects 2 and 6

These projects contained substantial structural differences from the Consortium CDEs, including variations in insurance, race, household income, and comorbidity questions. Consequently, one-to-many, and many-to-many mappings were common, and frequently required researcher judgment. Although overall semantic group accuracy remained relatively high, incomplete mappings were common because multiple harmonization strategies were possible. For example, the Consortium CDE included items on heart disease diagnosis and its treatment, which asked about the current diagnosis and treatment of heart diseases. In contrast, the corresponding Project 2 questions asked about the current and past diagnosis of heart attack and the current and past treatment for heart failure (heart attacks and heart failure are related but not the same). Similarly, questions about kidney disease and lung disease did not explicitly ask about treatment or functional limitations from diseases like Consortium CDEs but included treatment in the disease item description that indirectly suggested functional limitations, such as hemodialysis, peritoneal dialysis, and kidney transplantation. Variable response mapping also required additional review when response categories differed between the project and CDE formats. As presented in Table S3 in Multimedia Appendix 4, the semantically mapped race items were classified as incomplete mappings due to differences in question logic, demonstrating difficulties in harmonizing race obtained from different question structures.

#### Projects 3, 6, and 8

Subjective constructs measured using different validated instruments, including depression, stress, and loneliness, were particularly challenging for fully automated mapping. The LLM often omitted these items when the questionnaire wording differed from the Consortium CDEs (ie, depression measured with Patient Health Questionnaire vs PSS), even when the underlying constructs were conceptually related. In these cases, HITL review successfully identified additional candidate mappings but researcher judgment remained necessary because complete harmonization depended on study-specific analytic objectives.

#### Projects 4, 5, and 7

These projects largely consisted of questionnaire items corresponding to the Consortium CDEs and achieved semantic group accuracies of 98%‐100%. Nevertheless, subtle differences in branching logic, response options, and question structure still required manual review. Examples include multiple-choice versus single-choice insurance questions, household income collected using federal poverty level threshold categories rather than income ranges, and repeated one-to-many mappings for social needs questions.

## Discussion

### Principal Findings

This proof-of-concept study demonstrates that successful LLM-assisted DH depends on the design of the overall workflow rather than on the LLM alone. Applied to 8 Consortium projects, the pipeline successfully integrated LLMs into semantic mapping, variable response mapping, and data transformation while preserving researcher oversight throughout the harmonization process. The relatively high mapping accuracy observed in this study was facilitated, in part, by the Consortium’s bidirectional harmonization framework, in which the Consortium CDEs were developed through an iterative consensus process involving community research centers, the Research Coordinating Center, and other stakeholders.

### Value of HITL

Although semantic mapping accuracy was high both with and without HITL review, researcher involvement consistently reduced omission errors and incorrect mappings while producing more consistent performance across projects. Notably, omission of valid semantic mappings occurred more frequently than incorrect semantic mappings. These omissions were most common when variables required one-to-many or many-to-many mappings or when conceptually similar constructs were measured using different questionnaires. Subjective constructs, including depression, stress, and loneliness, were particularly challenging because different validated instruments measured similar concepts using different wording and response structures. These findings suggest that human review should be prioritized for variables with heterogeneous response formats and subjective constructs rather than uniformly applied across all semantic mappings. The need for HITL is likely to decrease as participating studies adopt standardized CDEs prospectively after the Consortium CDE’s launch, although researcher oversight will probably remain necessary for heterogeneous response structures and subjective constructs.

### Pipeline Design Matters

Our findings demonstrate that successful LLM-assisted harmonization depends not only on model capability but also on thoughtful pipeline design. Providing clear and specific instructions to the LLM reduced errors during both semantic and variable response mapping. Dividing large data dictionaries into concept-based chunks, providing complete metadata (including section headers and branching logic), and incorporating explicit verification steps into prompts all improved mapping accuracy and reduced omissions. Similarly, restructuring metadata during preprocessing enabled the LLM to better distinguish questionnaire instructions from survey items, highlighting the importance of data preparation in addition to prompt engineering.

### Human Factors Matter

Our experience also showed that successful workflow design must account for human cognitive workload in addition to LLM performance. Although we initially explored a fully automated workflow, an iterative HITL review approach proved more practical. Fully automated approaches generated comprehensive outputs but also increased computational cost, development time, and the cognitive burden associated with reviewing lengthy LLM responses. As the output volume increased, researchers were more likely to overlook omitted or incorrect mappings despite repeated review. This observation is consistent with previous studies suggesting that overreliance on AI-generated recommendations can adversely affect human decision making when AI outputs contain systematic errors [41,42]. Consequently, dividing harmonization into smaller, verifiable steps was more effective than attempting end-to-end automation. In our experience, rather than replacing human expertise, LLMs were more effective when supporting researchers in a structured, iterative workflow. This human-centered approach is also consistent with the Belmont Principles of beneficence, respect for persons, and justice, that emphasize responsible use of AI in research [43,44].

### Implications for Future Research Consortia

Our findings also have implications for the design of future multicenter research consortia. Establishing data governance policies, standardized CDEs, and harmonization expectations at study initiation can substantially reduce semantic heterogeneity and facilitate downstream DH [19]. However, prospective standardization alone is unlikely to eliminate the need for HITL review. Study-specific adaptations, evolving research objectives, and differences in questionnaire implementation can introduce semantic ambiguity that requires domain expertise to resolve. In our experience, conceptually complex variables, response value mappings, and context-dependent questionnaire items were more likely to benefit from expert review. Rather than replacing human expertise, LLMs may be most effective as decision-support tools that accelerate harmonization while allowing investigators to focus on the relatively small number of challenging mappings requiring expert consensus.

### Limitations

This proof-of-concept study has several limitations. First, the workflow simplified several aspects of real-world DH. For example, missing data mechanisms and differences arising from branching logic or unavailable response categories were simplified for this evaluation. Second, although this AI-implemented pipeline reduced the manual effort required for semantic mapping and variable transformation, actual consortium-wide harmonization also involves substantial time for data sharing, updating data dictionaries, communication among investigators, and consensus building, which were not fully represented in this study [9].

Third, this study included 8 projects selected as a proof of concept, whereas the Consortium ultimately comprises 41 projects. As the number and diversity of participating projects increase, semantic heterogeneity and harmonization complexity are also expected to increase. Although prospective data governance and standardized CDEs can facilitate harmonization, the high mapping accuracy observed in this study should not be generalized to larger or more heterogeneous harmonization efforts without careful consideration.

Finally, this study did not evaluate the environmental impact or long-term sustainability of LLM-assisted harmonization. Further research should examine how to balance the efficiency gains proved by LLMs with their computational costs and broader sustainability implications [45-47].

### Conclusion

This proof-of-concept study demonstrates that an LLM-assisted, HITL DH workflow can improve the efficiency of harmonizing heterogeneous research data while maintaining high semantic mapping accuracy. Rather than replacing researcher expertise, LLMs were most effective when integrated into a structured workflow that combined semantic mapping, variable response mapping, iterative verification, and human oversight.

These findings provide practical guidance for researchers seeking to incorporate LLMs into DH workflows, particularly when harmonizing questionnaire-based data and SDOH CDEs. Our results also highlight that careful preprocessing, prompt design, and targeted human review are essential for handling heterogeneous response structures and subjective constructs measured using different instruments.

Future research should evaluate the scalability of this approach in larger and more heterogeneous research consortia, refine harmonization strategies for complex variable structures, and develop transparent, reproducible workflows for LLM-assisted DH. Such efforts will help maximize the value of harmonized research data for secondary analyses while supporting the responsible, and human-centered use of AI in biomedical research.

## Supplementary material

10.2196/93996 Multimedia Appendix 1 CDE concepts, question items, and semantic groups

10.2196/93996 Multimedia Appendix 2 Details of the included studies

10.2196/93996 Multimedia Appendix 3 Mapping Results of the Required CDE Semantic Groups

10.2196/93996 Multimedia Appendix 4 Analysis for the incomplete mapping items.

10.2196/93996 Multimedia Appendix 5 Example R code LLM generated for variable mapping.

## Abbreviations

AUROC: area under the receiver operating characteristic curve
BERT: Bidirectional Encoder Representations from Transformers
BioSHaRE: Biobank Standardization and Harmonization for Research Excellence in the European Union
CDE: Common Data Element
CDM: Common Data Model
DH: data harmonization
DIVER: Data Inventory and Verification Environment for Research
EHR: electronic health record
GPT: Generative Pretrained Transformer
HIPAA: Health Insurance Portability and Accountability Act
HITL: human-in-the-loop
*ICD-10*: *International Statistical Classification of Diseases, Tenth Revision*
LLM: large language model
MCD-DR: Multiple Chronic Disease Disparities Research
MENA: Middle Eastern and North African
NIH: National Institutes of Health
NIHMD: National Institute on Minority Health and Health Disparities
NLP: natural language processing
OMOP: Observational Medical Outcomes Partnership
PCORI: Patient-Centered Outcomes Research Institute
PCORnet: National Patient-Centered Clinical Research Network
PhenX: Phenotypes and eXposures
PROMIS: Patient-Reported Outcomes Measurement Information System
UCSF: University of California San Francisco
