# Supplementary material for: Large Language Model–Assisted Semiautomated Data Harmonization Pipeline for the Multiple Chronic Disease Disparities Research Consortium: Framework Development and Comparative Validation Study
Source: JMIR AI. 2026 Oct 22;5:e93996. doi: 10.2196/93996 (PMC13690333; doi:10.2196/93996)
Supplement: Supplementary file 1 [file ai-v5-e93996-s001.docx]

**Table S1.** CDE concepts, question items, and semantic groups

| Item number | CDE question item  (developed by the Working Group) | CDE concepts   (developed by the Working Group) | CDE semantic group  (developed for this study) |  |
| --- | --- | --- | --- | --- |
|  |  |  |  |  |
| 2 | ethnicity | Ethnicity | Ethnicity |  |
| 3 | ethnicity_hispanic |  |  |  |
| 4 | ethnicity_other |  |  |  |
| 5 | race | Race | Race |  |
| 6 | race_otr |  |  |  |
| 7 | sex_assigned_at_birth | Sex assigned at birth | Sex assigned at birth |  |
| 8 | sex_assigned_at_birth_o_2 |  |  |  |
| 9 | gender_identity_term | Gender identity term | Gender identity term |  |
| 10 | gender_identity_description_o |  |  |  |
| 11 | sexual_orientation_identity | Sexual orientation | Sexual orientation |  |
| 12 | sexual_orientation_description_o |  |  |  |
| 13 | age_in_years | Age in years | Age in years |  |
| 14 | age_in_years_no_response |  |  |  |
| 18 | fi_12_mos_food_money_freq | Food insecurity  (2 items among 18-29, 22, 23 are required) | Food insecurity 1 |  |
| 19 | fi_12_mos_afford_balanced_meals |  | Food insecurity 2 |  |
| 20 | fi_12_mos_change_diet |  | Food insecurity 3 |  |
| 21 | fi_change_diet_frequency |  | Food insecurity 4 |  |
| 22 | fi_12_mos_eat_less |  | Food insecurity 5 |  |
| 23 | fi_12_mos_hungry |  | Food insecurity 6 |  |
| 25 | edu_att_individual_highest_grade | Education attainment | Education attainment |  |
| 26 | health_literacy_medical_forms | Health literacy | Health literacy |  |
| 29 | ann_family_inc_household | Household size | Household size |  |
| 32 | ann_family_inc_total_last_yr | Household income and categories (either household income or income category question is required) | Household income |  |
| 33 | ann_family_inc_total_last_yr_enc |  |  |  |
| 35 | ann_family_inc_total_thld_250 |  | Household income subcategory 1 |  |
| 37 | ann_family_inc_total_thld_138 |  | Household income subcategory 2 |  |
| 39 | ann_family_inc_total_thld_100 |  | Household income subcategory 3 |  |
| 41 | ann_family_inc_total_thld_200 |  | Household income subcategory 4 |  |
| 42 | annual_family_income_total_75 |  | Household income subcategory 5 |  |
| 43 | annual_family_income_total_100 |  | Household income subcategory 6 |  |
| 45 | ann_family_inc_total_thld_400 |  | Household income subcategory 7 |  |
| 46 | annual_family_income_total_150 |  | Household income subcategory 8 |  |
| 49 | ann_fam_inc_2022fpgcats |  | Household income category |  |
| 50 | current_employment_status | Current employment status | Current employment status |  |
| 51 | cur_employ_stat_specify |  |  |  |
| 52 | ahc_hrsn_st_suppl_edu_q15 | Foreign language at home | Foreign language at home |  |
| 53 | english_proficiency_speak_engl | English proficiency | English proficiency |  |
| 54 | acs_hlth_svcs_last_seen_doctor | Last seen doctor | Last seen doctor |  |
| 55 | acs_hlth_svcs_usual_place_hc | Usual place for health care | Usual place for health care |  |
| 56 | acs_hlth_svcs_hc_most_often | Most often place for health care | Most often place for health care |  |
| 57 | acs_hlth_svcs_hc_most_often_o |  |  |  |
| 58 | acs_hlth_svcs_past_12_mos_uc | Urgent care visit for past year | Urgent care visit for past year |  |
| 59 | acs_hlth_svcs_past_12_mos_uc_dk |  |  |  |
| 60 | acs_hlth_svcs_past_12_mos_uc_v |  |  |  |
| 61 | acs_hlth_svcs_past_12_mos_er | Emergency visit for past year | Emergency visit for past year |  |
| 62 | acs_hlth_svcs_past_12_mos_er_dk |  |  |  |
| 63 | acs_hlth_svcs_past_12_mos_er_v |  |  |  |
| 64 | acs_hlth_svcs_delayed_mc | Delayed medical care | Delayed medical care |  |
| 65 | hlth_ins_coverage_employer | Health insurance | Health insurance |  |
| 66 | 50hlth_ins_coverage_purchased |  |  |  |
| 67 | hlth_ins_coverage_medicaid |  |  |  |
| 68 | hlth_ins_coverage_medicare |  |  |  |
| 69 | hlth_ins_coverage_military |  |  |  |
| 70 | hlth_ins_coverage_indian |  |  |  |
| 71 | hlth_ins_coverage_other |  |  |  |
| 72 | hlth_ins_coverage_nocoverage |  |  |  |
| 73 | hlth_ins_coverage_f1adcd |  |  |  |
| 74 | cls_decription | Current living situation | Current living situation |  |
| 75 | cls_description_other |  |  |  |
| 76 | 60cls_trouble_paying_food | Economic stability – social needs | Social needs |  |
| 77 | cls_trouble_paying_housing |  |  |  |
| 78 | cls_trouble_paying_heat_electric |  |  |  |
| 79 | cls_trouble_paying_medical |  |  |  |
| 80 | cls_trouble_paying_transport |  |  |  |
| 81 | cls_trouble_paying_childcare |  |  |  |
| 82 | cls_trouble_paying_debts |  |  |  |
| 83 | trouble_paying_phone |  |  |  |
| 84 | trouble_paying_internet |  |  |  |
| 85 | cls_trouble_paying_none |  |  |  |
| 86 | 70cls_trouble_paying_o |  |  |  |
| 87 | cls_other_text |  |  |  |
| 88 | cls_lack_of_transport_m_appts | Lack of access to transportation | Lack of access to transportation |  |
| 89 | cls_lack_of_transport_m_adl |  |  |  |
| 90 | cls_lack_of_transport_m_prob |  |  |  |
| 91 | cls_relationship_status | Current relationship status | Current relationship status |  |
| 92 | cls_hard_get_medication | Hard to get medication | Hard to get medication |  |
| 93 | cls_need_help_to_read | Literacy | Literacy |  |
| 94 | cls_lonely | Loneliness | Loneliness |  |
| 95 | cls_social_connection | Social connection | Social connection |  |
| 96 | 80ahc_hrsn_st_suppl_fcs_q13 | Daily help | Daily help |  |
| 97 | ahc_hrsn_st_suppl_su_q19 | Alcohol use | Alcohol use |  |
| 98 | ahc_hrsn_st_suppl_su_q19_male |  |  |  |
| 99 | ahc_hrsn_st_suppl_su_q19_female |  |  |  |
| 100 | path_lifetime_tobacco_use | Lifetime tobacco use | Lifetime tobacco use |  |
| 101 | path_tobacco_use_last_year | Last year tobacco use | Last year tobacco use |  |
| 102 | path_tobacco_use_30_days | Last month tobacco use | Last month tobacco use |  |
| 103 | path_tobacco_use_times_30_days | Frequency of last month tobacco use | Frequency of last month tobacco use |  |
| 104 | ahc_hrsn_st_suppl_pa_q17 | Moderate exercise days in past month | Moderate exercise days in past month |  |
| 105 | ahc_hrsn_st_suppl_pa_q18 | Moderate exercise duration (minutes) | Moderate exercise duration (minutes) |  |
| 106 | ahc_hrsn_st_suppl_mh_q23b | Depression | Depression |  |
| 107 | ahc_hrsn_st_suppl_mh_q24 | Stress | Stress |  |
| 108 | disability_mental | Disability in cognition | Disability in cognition |  |
| 109 | disability_errands | Disability in errands | Disability in errands |  |
| 110 | global_03 | Quality of life | Quality of life |  |
| 112 | comorbid | Comorbidity | Comorbidity_EHR |  |
| 116 | scq_covid19 |  | Comorbidity dx 1 |  |
| 117 | scq_heart |  | Comorbidity dx 2 |  |
| 118 | scq_hbp |  | Comorbidity dx 3 |  |
| 119 | scq_lung |  | Comorbidity dx 4 |  |
| 120 | scq_diabetes |  | Comorbidity dx 5 |  |
| 121 | scq_stomach |  | Comorbidity dx 6 |  |
| 122 | scq_kidney |  | Comorbidity dx 7 |  |
| 123 | scq_liver |  | Comorbidity dx 8 |  |
| 124 | scq_blood |  | Comorbidity dx 9 |  |
| 125 | scq_cancer |  | Comorbidity dx 10 |  |
| 126 | scq_depression |  | Comorbidity dx 11 |  |
| 127 | scq_osteoarthritis |  | Comorbidity dx 12 |  |
| 128 | scq_backpain |  | Comorbidity dx 13 |  |
| 129 | scq_ra |  | Comorbidity dx 14 |  |
| 130 | scq_trtmt_covid19 |  | Comorbidity tx 1 |  |
| 132 | scq_trtmt_heart |  | Comorbidity tx 2 |  |
| 133 | scq_trtmt_hbp |  | Comorbidity tx 3 |  |
| 134 | scq_trtmt_lung |  | Comorbidity tx 4 |  |
| 135 | scq_trtmt_diabetes |  | Comorbidity tx 5 |  |
| 136 | scq_trtmt_stomach |  | Comorbidity tx 6 |  |
| 137 | scq_trtmt_kidney |  | Comorbidity tx 7 |  |
| 138 | scq_trtmt_liver |  | Comorbidity tx 8 |  |
| 139 | scq_trtmt_blood |  | Comorbidity tx 9 |  |
| 140 | scq_trtmt_cancer |  | Comorbidity tx 10 |  |
| 141 | scq_trtmt_depression |  | Comorbidity tx 11 |  |
| 142 | scq_trtmt_osteoarthritis |  | Comorbidity tx 12 |  |
| 143 | scq_trtmt_backpain |  | Comorbidity tx 13 |  |
| 144 | scq_trtmt_ra |  | Comorbidity tx 14 |  |
| 146 | scq_limit_covid19 |  | Comorbidity fx 1 |  |
| 147 | scq_limit_heart |  | Comorbidity fx 2 |  |
| 148 | scq_limit_hbp |  | Comorbidity fx 3 |  |
| 149 | scq_limit_lung |  | Comorbidity fx 4 |  |
| 150 | scq_limit_diabetes |  | Comorbidity fx 5 |  |
| 151 | scq_limit_stomach |  | Comorbidity fx 6 |  |
| 152 | scq_limit_kidney |  | Comorbidity fx 7 |  |
| 153 | scq_limit_liver |  | Comorbidity fx 8 |  |
| 154 | scq_limit_blood |  | Comorbidity fx 9 |  |
| 155 | scq_limit_cancer |  | Comorbidity fx 10 |  |
| 156 | scq_limit_depression |  | Comorbidity fx 11 |  |
| 157 | scq_limit_osteoarthritis |  | Comorbidity fx 12 |  |
| 158 | scq_limit_backpain |  | Comorbidity fx 13 |  |
| 159 | scq_limit_ra |  | Comorbidity fx 14 |  |
| 162 | birthplace | Birthplace | Birthplace 1 |  |
| 163 | birthplace_location_in_us |  | Birthplace 2 |  |
| 164 | birthplace_location_in_us_terr |  | Birthplace 3 |  |
| 165 | birthplace_foreign_country |  | Birthplace 4 |  |
| 166 | birthplace_foreign_country_o |  |  |  |
| 167 | cls_interpersonal_violence | Interpersonal violence | Interpersonal violence |  |
| 168 | cls_ip_violence_specify |  |  |  |
| 169 | cls_financial_abuse | Financial abuse | Financial abuse |  |
| 170 | brfss_marijuana_m21_01 | Marijuana use | Marijuana use |  |
| 171 | ahc_hrsn_st_suppl_su_q21 | Drug abuse | Drug abuse |  |
| 172 | ahc_hrsn_st_suppl_su_q22 | Illicit drug use | Illicit drug use |  |
| 173 | perceived_discrim_qx | Perceived discrimination in medical care | Perceived discrimination in medical care |  |
| 174 | disability_deaf | Deaf | Deaf |  |
| 175 | disability_blind | Blind | Blind |  |
| 176 | disability_walking | Disability in walking | Disability in walking |  |
| 177 | disability_dressing | Disability in dressing | Disability in dressing |  |
| 178 | disability_communicate | Disability in communicate | Disability in communicate |  |

**Abbreviations:** CDE = Common Data Element, dx = diagnosis, fx = function, tx = treatment

**Note:** CDE questions were selected from established instruments and national toolkits, including the PhenX Toolkit, PROMIS Global Health, the Accountable Health Communities Health-Related Social Needs Screening Tool, and the All of Us Research Program SDOH Module. Additional information on the Multiple Chronic Disease Disparities Research Consortium CDEs is publicly available on the website (https://health-excellence-action.org/nih-cde).
