# Supplementary material for: Large Language Model–Assisted Semiautomated Data Harmonization Pipeline for the Multiple Chronic Disease Disparities Research Consortium: Framework Development and Comparative Validation Study
Source: JMIR AI. 2026 Oct 22;5:e93996. doi: 10.2196/93996 (PMC13690333; doi:10.2196/93996)
Supplement: Supplementary file 2 [file ai-v5-e93996-s002.docx]

**Table S2.** Details of the included studies

| # | Study design | Study population | Intervention  /Exposure | Control | Outcome | Launch before the final CDE development | Domains | | | | | | | | |
| --- | --- | --- | --- | --- | --- | --- | --- | --- | --- | --- | --- | --- | --- | --- | --- |
|  |  |  |  |  |  |  | Demo | Neighborhood | Food | Education | Economic | Social | Health care | Discrimination | Disability |
| 1 | RCT | Vietnamese and Spanish-speaking communities 5 years and older | Skills-based educational strategies for reduction of vascular events | Enhanced standard intervention | Life's Essential 8 | Yes | █ |  | █ | █ | █ | █ | █ |  |  |
| 2 | Sequential cluster RCT | African American adults 40 years of age and older | Multi-component PA education program | Go 4 Life Self-Guided Education | Changes in physical function | Yes | █ | █ | █ | █ | █ | █ | █ | █ | █ |
| 3 | RCT | Black adults 40–70 years of age | Intensive lifestyle intervention | Usual care | Changes in body weight | Yes | █ | █ | █ | █ | █ | █ | █ |  | █ |
| 4 | RCT | Breast cancer patients aged 18–99 years who has been prescribed endocrine therapy | Multicomponent adherence intervention | Usual care | Number of participants adherent to endocrine therapy and cardiovascular disease medication at 24 Weeks | Yes | █ | █ | █ | █ | █ | █ | █ | █ | █ |
| 5 | Not specified | Adults with hypertension and type 2 diabetes | Food-is-Medicine intervention | Not specified | Not specified | No | █ | █ | █ | █ | █ | █ | █ |  | █ |
| 6 | RCT | Black/African Americans 18 years and older | Community health worked based intervention and antihypertensive medication | Usual care | Changes in systolic blood pressure and NT-proB-type Natriuretic Peptide levels over 12 months | Yes | █ | █ | █ | █ | █ | █ | █ |  | █ |
| 7 | RCT | Latino children ages 5 to 12 years of age | Parent-based treatment- All caregivers | Health Education | Child anthropometric change as measured by age and sex adjusted body mass index | Yes | █ | █ | █ | █ | █ | █ | █ |  | █ |
| 8 | RCT | 50 Years and older spouse or other family caregiver of Alzheimer's disease and related dementias with Chinese or Korean ancestry | Counseling sessions, participation in local support group and on-line chat group, and ‘ad hoc' counseling | Participation in an online chat group, and call the counselor for resource information and support as needed | Depressive symptoms | Yes | █ | █ |  | █ | █ | █ | █ |  |  |

**Abbreviations and symbols:** AAC = Ask-advice-connect, CHD = coronary heart disease, Demo = demographics, Economic = income/employment/financial strain, Education = education attainment & health literacy, Food = food insecurity, Health Care = health and clinical care/access, Neighborhood = neighborhood & physical environment (including transportation), PA = physical activity, RCT = randomized controlled trials, Social = social & community context, # = project number, █ = the project included one or more question items representing the corresponding domain.
