# Supplementary material for: Large Language Model–Assisted Semiautomated Data Harmonization Pipeline for the Multiple Chronic Disease Disparities Research Consortium: Framework Development and Comparative Validation Study
Source: JMIR AI. 2026 Oct 22;5:e93996. doi: 10.2196/93996 (PMC13690333; doi:10.2196/93996)
Supplement: Supplementary file 3 [file ai-v5-e93996-s003.docx]

**Table S3.** Mapping Results of the Required CDE Semantic Groups

|  | **Project 1** | **Project 2** | **Project 3** | **Project 4** | **Project 5** | **Project 6** | **Project 7** | **Project 8** |
| --- | --- | --- | --- | --- | --- | --- | --- | --- |
| **Demographics and social determinants of health variables 1** | | | | | | | | |
| Ethnicity |  |  |  |  |  |  |  |  |
| Race |  |  |  |  |  |  |  |  |
| Sex at birth |  |  |  |  |  |  |  |  |
| Gender identity |  |  |  |  |  |  |  |  |
| Sexual orientation |  |  |  |  |  |  |  |  |
| Age |  |  |  |  |  |  |  |  |
| Marital status |  |  |  |  |  |  |  |  |
| Literacy |  |  |  |  |  |  |  |  |
| Education |  |  |  |  |  |  |  |  |
| Health literacy |  |  |  |  |  |  |  |  |
| Foreign language |  |  |  |  |  |  |  |  |
| English proficiency |  |  |  |  |  |  |  |  |
| Loneliness |  |  |  |  |  |  |  |  |
| Social connection |  |  |  |  |  |  |  |  |
| Depression |  |  |  |  |  |  |  |  |
| Stress |  |  |  |  |  |  |  |  |
| General physical health |  |  |  |  |  |  |  |  |
| Mental disability |  |  |  |  |  |  |  |  |
| Disability errands |  |  |  |  |  |  |  |  |
| Daily help |  |  |  |  |  |  |  |  |
| Alcohol use |  |  |  |  |  |  |  |  |
| Lifetime smoking |  |  |  |  |  |  |  |  |
| Past year smoking |  |  |  |  |  |  |  |  |
| Past month smoking |  |  |  |  |  |  |  |  |
| Past month smoking frequency |  |  |  |  |  |  |  |  |
| Moderate exercise frequency |  |  |  |  |  |  |  |  |
| Moderate exercise time |  |  |  |  |  |  |  |  |
| **Social determinants of health variables 2** | | | | | | | | |
| Food security 1 |  |  |  |  |  |  |  |  |
| Food security 2 |  |  |  |  |  |  |  |  |
| Food security 3 |  |  |  |  |  |  |  |  |
| Food security 4 |  |  |  |  |  |  |  |  |
| Food security 5 |  |  |  |  |  |  |  |  |
| Food security 6 |  |  |  |  |  |  |  |  |
| Household size |  |  |  |  |  |  |  |  |
| Household income |  |  |  |  |  |  |  |  |
| Household income range |  |  |  |  |  |  |  |  |
| Employment |  |  |  |  |  |  |  |  |
| Last seen doctor |  |  |  |  |  |  |  |  |
| Usual place of care |  |  |  |  |  |  |  |  |
| Most often place of care |  |  |  |  |  |  |  |  |
| Urgent care |  |  |  |  |  |  |  |  |
| Emergency care |  |  |  |  |  |  |  |  |
| Delayed care |  |  |  |  |  |  |  |  |
| Insurance |  |  |  |  |  |  |  |  |
| Living situation |  |  |  |  |  |  |  |  |
| Social need |  |  |  |  |  |  |  |  |
| Hard to get medication |  |  |  |  |  |  |  |  |
| Lack of transportation |  |  |  |  |  |  |  |  |
| **Comorbidity-related diagnosis variables** | | | | | | | | |
| Ever COVID |  |  |  |  |  |  |  |  |
| Heart disease |  |  |  |  |  |  |  |  |
| HBP |  |  |  |  |  |  |  |  |
| Lung disease |  |  |  |  |  |  |  |  |
| Diabetes mellitus |  |  |  |  |  |  |  |  |
| Stomach disease |  |  |  |  |  |  |  |  |
| Kidney disease |  |  |  |  |  |  |  |  |
| Liver disease |  |  |  |  |  |  |  |  |
| Blood disease |  |  |  |  |  |  |  |  |
| Cancer |  |  |  |  |  |  |  |  |
| Depression |  |  |  |  |  |  |  |  |
| Osteoarthritis |  |  |  |  |  |  |  |  |
| Back pain |  |  |  |  |  |  |  |  |
| Rheumatoid arthritis |  |  |  |  |  |  |  |  |
| **Comorbidity-related treatment variables** | | | | | | | | |
| Ever COVID |  |  |  |  |  |  |  |  |
| Heart disease |  |  |  |  |  |  |  |  |
| HBP |  |  |  |  |  |  |  |  |
| Lung disease |  |  |  |  |  |  |  |  |
| Diabetes mellitus |  |  |  |  |  |  |  |  |
| Stomach disease |  |  |  |  |  |  |  |  |
| Kidney disease |  |  |  |  |  |  |  |  |
| Liver disease |  |  |  |  |  |  |  |  |
| Blood disease |  |  |  |  |  |  |  |  |
| Cancer |  |  |  |  |  |  |  |  |
| Depression |  |  |  |  |  |  |  |  |
| Osteoarthritis |  |  |  |  |  |  |  |  |
| Back pain |  |  |  |  |  |  |  |  |
| Rheumatoid arthritis |  |  |  |  |  |  |  |  |
| **Comorbidity-related functional limitation variables** | | | | | | | | |
| Ever COVID |  |  |  |  |  |  |  |  |
| Heart disease |  |  |  |  |  |  |  |  |
| HBP |  |  |  |  |  |  |  |  |
| Lung disease |  |  |  |  |  |  |  |  |
| Diabetes mellitus |  |  |  |  |  |  |  |  |
| Stomach disease |  |  |  |  |  |  |  |  |
| Kidney disease |  |  |  |  |  |  |  |  |
| Liver disease |  |  |  |  |  |  |  |  |
| Blood disease |  |  |  |  |  |  |  |  |
| Cancer |  |  |  |  |  |  |  |  |
| Depression |  |  |  |  |  |  |  |  |
| Osteoarthritis |  |  |  |  |  |  |  |  |
| Back pain |  |  |  |  |  |  |  |  |
| Rheumatoid arthritis |  |  |  |  |  |  |  |  |

Abbreviations and symbols: CDE = Common Data Element, HBP = high blood pressure, = complete variable mapping, = incomplete variable mapping, = incomplete semantic mapping, = not available

Note: The household income subcategories and the electronic health record comorbidity semantic group were excluded. For household income, projects could collect either the household income or household income range variable.
