# Supplementary material for: Large Language Model–Assisted Semiautomated Data Harmonization Pipeline for the Multiple Chronic Disease Disparities Research Consortium: Framework Development and Comparative Validation Study
Source: JMIR AI. 2026 Oct 22;5:e93996. doi: 10.2196/93996 (PMC13690333; doi:10.2196/93996)
Supplement: Supplementary file 4 [file ai-v5-e93996-s004.docx]

**Table S4.** Analysis for the incomplete mapping items

| **Example 1. Questions about the current condition and those about past or current conditions were matched** | |
| --- | --- |
| CDE  Do you have the problem?  COVID-19 (SARS-Cov2) -- ever tested positive  1, Yes \| 0, No | Project  Do you have the problem?  COVID-19  1, Yes \| 0, No |
| **Example 2. Questions that need to be mapped to other comorbidity were not matched** | |
| CDE  Please write in any other medical problems that you have. | Project  Have you been diagnosed with hyperlipidemia  1, Yes \| 0, No \| 2, Don't know/ Unsure |
| **Example 3. Questions that need consensus on where to map** | |
| CDE  Do you have the problem?  Heart disease  1, Yes \| 0, No  Do you receive treatment for it?  Heart disease  1, Yes \| 0, No | Project  Have you ever had a heart attack?  1, Yes \| 0, No \| 2, Don't know/ Unsure  Have you ever been treated for heart failure? (You may have been short of breath and the doctor may have told you that you had fluid in your lungs or that your heart was not pumping well.)  1, Yes \| 0, No \| 2, Don't know/ Unsure |
| **Example 4. Questions LLM mapped** | |
| CDE  Because of a physical, mental, or emotional condition, do you have serious difficulty concentrating, remembering, or making decisions? | Project  Anxiety / depression  1, I am not anxious or depressed \| 2, I am slightly anxious or depressed \| 3, I am moderately anxious or depressed \| 4, I am severely anxious or depressed \| 5, I am extremely anxious or depressed \| -99, skip |
| **Example 5. Need consensus on how to map the response options** | |
| CDE  Are you currently covered by any of the  following types of health insurance or health coverage plans?  Insurance through a current or former  employer or union (of yours or another family  member's). This would include COBRA coverage.  Insurance purchased directly from an insurance company (by you or another family member). This would include coverage purchased through an exchange or marketplace, such as HealthCare.gov [if the respondent is in a state with state-specific names, insert] [or insert program name]  Medicaid, Medical Assistance (MA), the Children's Health Insurance Program (CHIP), or any kind of state or government-sponsored assistance plan based on income or disability. You may know this type of coverage as [if the respondent is in a state with state-specific names insert program name].  Medicare, for people 65 and older, or people with certain disabilities  TRICARE or other military health care, including VA health care  Indian Health Service  Any Other type of health insurance coverage or health coverage plan?  Can choose options below for questions above  1, Covered \| 2, Not covered \| 3, Not sure  Does this mean you currently have no health insurance or health coverage plan? In answering this question, please exclude plans that pay for only one type of service (such as, nursing home care, accidents, family planning, or dental care) and plans that only provide extra cash when hospitalized.  1, do NOT have health insurance \| 2, HAVE some kind of health insurance  What type of health insurance do you have?  Fill in text | Project  What type of health insurance do you have?  1, Private insurance \| 2, Medicare \| 3, Medicare and private insurance \| 4, Medicaid \| 5, Medicaid managed care \| 6, Medicaid and Medicare \| 7, Military or veterans sponsored NOS \| 8, Military Sponsored (Including CHAMPUS and TriCare) \| 9, Veterans sponsored \| 10, Indian health service \| 11, Self-pay (No insurance) \| 12, No means of payment (No health insurance or health coverage plan) \| 13, Other, please specify: |
| **Example 6. Differences in response categories** | |
| CDE  What is your best estimate of the total income of all family members from all sources, before taxes, in the last calendar year?  1, less than $13,590 \| 2, $13,590 to $18,309 \| 3, $18,310 to $23,029 \| 4, $23,030 to $27,749 \| 5, $27,750 to $32,469 \| 6, $32,470 to $37,189 \| 7, $37,190 to $41,909 \| 8, $41,910 to $46,629 \| 9, $46,630 to $51,349 \| 10, $51,350 to $56,069 \| 11, $56,070 to $60,789  \| 12, $60,790 to $65,509 \| 13, $65,510 to $70,229 \| 14, $70,230 to $74,949 \| 15, $74,950 to $79,669 \| 16, $79,670 to $84,389 \| 17, $84,390 to $89,109 \| 18, $89,110 to $93,829 \| 19, $93,830 to $98,549 \| 20, $98,550 to $103,269 \| 21, more than $103,269 \| -77, Don't know \| -88, Prefer not to answer | Project  What was the total combined income of your household in the past year, including income from all sources such as wages, salaries, Social Security or retirement benefits, help from relatives and so forth? Please tell us the total income before taxes.  1, Under $10,000 \| 2, $10,000 to $14,999 \| 3, $15,000 to $19,999 \| 4, $20,000 to $29,999 \| 5, $30,000 to $39,999 \| 6, $40,000 to $49,999 \| 7, $50,000 to $59,999 \| 8, $60,000 to $69,999 \| 9, $70,000 to $79,999 \| 10, $80,000 to $89,999 \| 11, $90,000 to $99,999 \| 12, $100,000 or above \| -77, I don't know \| -88, Prefer not to answer |
| **Example 7. Differences in branching logic** | |
| Same question above | Project (question, branching logic)  Is your total household income below $13,590? [hh_ppl]='1'  Is your total household income below $18,310? [hh_ppl]='2'  Is your total household income below $23,030? [hh_ppl]='3'  $84,390? [hh_ppl]='16'  …(omitted)...  Is your total household income below $93,830? [hh_ppl] ='18'  Is your total household income below $98,550? [hh_ppl] ='19'  Is your total household income below $103,270? [hh_ppl] ='20' |
| **Example 8. Differences in question logic (single-choice vs. multiple-choice)** | |
| CDE  What is your race? (Check all that apply)  1, American Indian or Alaska Native \| 2, Asian \| 3, Black or African American \| 4, Native Hawaiian or Other Pacific Islander \| 5, North African, Middle Eastern, or Southwest Asian  \| 6, White \| 7, Some other race \| -88, Prefer not to answer  Detailed examples of response options are omitted here | Project  Race (single-choice)  1, Afghanistani \| 2, African \| 3, African American \| 4, American Indian \| 5, American Indian or Alaska Native \| 6, Arab \| 7, Asian \| 8, Asian Indian \| 9, Bangladeshi \| 10, Black \| 11, Black or African American \| 12, Chinese \| 13, Chippewa Cree \| 14, Egyptian \| 15, European \| 16, French American Indian \| 17, Indonesian \| 18, Iranian \| 19, Irish \| 20, Korean \| 21, Mexican American Indian \| 22, Middle Eastern or North African \| 23, Native Hawaiian or Other Pacific Islander \| 24, Pakistani \| 25, Scottish \| 26, South American Indian \| 27, Spanish American Indian \| 28, Vietnamese \| 29, West Indian \| 30, White \| 31, White Earth \| 32, White Mountain \| 888, Other Race \| 999, Patient Declined |
| **Example 9. Differences in question response options which need judgment calls** | |
| CDE  Are you of Hispanic, Latino, Latina, or Spanish origin?  0, No, NOT of Hispanic, Latino, Latina, or Spanish origin \| 1, Yes, of Hispanic, Latino, Latina, or Spanish origin \| -88, Prefer not to answer | Project  Ethnicity  0, Not Hispanic or Latino \| 1, Central American \| 2, Cuban \| 3, Dominican \| 4, Hispanic or Latino/Spanish \| 5, Latin American/Latin, Latino \| 6, Mexican \| 7, Mexican American \| 8, Puerto Rican \| 9, Salvadoran \| 10, South American \| 11, South American Indian \| 12, Spaniard \| 13, Uruguayan \| 999, Patient Declined |
