# Supplementary material for: Large Language Model–Assisted Semiautomated Data Harmonization Pipeline for the Multiple Chronic Disease Disparities Research Consortium: Framework Development and Comparative Validation Study
Source: JMIR AI. 2026 Oct 22;5:e93996. doi: 10.2196/93996 (PMC13690333; doi:10.2196/93996)
Supplement: Supplementary file 5 [file ai-v5-e93996-s005.docx]

**Table S5.** Example R code LLM generated for variable mapping

**Example 1. Multiple to one variable mapping**

Project

- How often does anyone, including family and friends, insult or talk down on you?

1, Never | 2, Rarely | 3, Sometimes | 4, Fairly Often | 5, Frequently

- How often does anyone, including family and friends, physically hurt you?

1, Never | 2, Rarely | 3, Sometimes | 4, Fairly Often | 5, Frequently

- How often does anyone, including family and friends, scream or curse at you?

1, Never | 2, Rarely | 3, Sometimes | 4, Fairly Often | 5, Frequently

- How often does anyone, including family and friends, threaten you with harm?

1, Never | 2, Rarely | 3, Sometimes | 4, Fairly Often | 5, Frequently

CDE

- In the past 12 months, have you been physically or emotionally hurt or felt threatened by a current or former spouse/partner, a caregiver, or someone else you know?

1, Yes | 0, No | -88, Prefer not to answer

LLM
\# Define a function to transform project variables to cde variable

transform_to_cde <- function(physical, insult, shout, threat) {

  # If any of the abuse responses are greater than 1 (i.e., not 'Never'), mark as 'Yes'

  # Otherwise, 'No'

  if (any(physical > 1, insult > 1, shout > 1, threat > 1, na.rm = TRUE)) {

    return(1)  # Yes

  } else if (all(physical == 1, insult == 1, shout == 1, threat == 1, na.rm = TRUE)) {

    return(0)  # No

  } else {

    return(-88)  # Prefer not to answer or unassessed data

  }

}

(omitted)

**Example 2. One to multiple variables mapping**

Project

- If yes, what is causing the pain? (Mark all that apply)

1, Lupus (systemic lupus erythematosus) | 2, Polymyalgia rheumatica | 3, Osteoarthritis | 4, Rheumatoid Arthritis | 5, Other, specify: {muscular_issue_oth} | 6, Don't know/ Unsure

CDE

Do you have the problem?

- Osteoarthritis, degenerative arthritis

1, Yes | 0, No

- Rheumatoid arthritis

1, Yes | 0, No

LLM

\# Transform the data

transformed_data <- project %>%

  mutate(scq_osteoarthritis = if_else(grepl("3", muscular_issue_what), 1, 0))

transformed_data <- project %>%

  mutate(scq_ra = if_else(grepl("4", muscular_issue_what), 1, 0))

**Example 3. Multiple to multiple variables mapping**

Project (same branching logic with CDE)

- Are you currently covered by any of the following types of health insurance or health coverage plans? (select ALL that apply)

1, Insurance through a current or former employer or union (of yours or another family member's) This would include COBRA coverage. | 2, Insurance purchased directly from an insurance company (by you or another family member) This would include coverage purchased through an exchange or marketplace, such as HealthCare.gov or Covered California. | 3, Medicare, for people 65 and older, or people with certain disabilities. | 4, Medicaid, Medical Assistance (MA), the Children's Health Insurance Program (CHIP), or any kind of state or government-sponsored assistance plan based on income or a disability. You may know this type of coverage as Medi-Cal. | 5, TRICARE or other military health care, including VA health care. | 6, Indian Health Service | 7, Any other type of health insurance coverage or health coverage plan | 8, None of the above | 77, Don't know | 88, Prefer not to answer

- Please specify if you are covered by any other type of health insurance or health coverage plan.
- Does this mean you currently have no health insurance or health coverage plan? In answering this question, please exclude plans that pay for only one type of service (such as, nursing home care, accidents, family planning, or dental care) and plans that only provide extra cash when hospitalized.

1, I do not have health insurance | 2, I have some kind of health insurance

- What type of health insurance do you have? (text)

CDE

Are you currently covered by any of the following types of health insurance or health coverage plans?

- Insurance through a current or former employer or union (of yours or another family member's). This would include COBRA coverage

1, Covered | 2, Not covered | 3, Not sure

- Insurance purchased directly from an insurance company (by you or another family member). This would include coverage purchased through an exchange or marketplace, such as HealthCare.gov [if the respondent is in a state with state-specific names, insert] [or insert program name]

1, Covered | 2, Not covered | 3, Not sure

- Medicaid, Medical Assistance (MA), the Children's Health Insurance Program (CHIP), or any kind of state or government-sponsored assistance plan based on income or disability. You may know this type of coverage as [if the respondent is in a state with state-specific names insert program name]

1, Covered | 2, Not covered | 3, Not sure

- Medicare, for people 65 and older, or people with certain disabilities

1, Covered | 2, Not covered | 3, Not sure

- TRICARE or other military health care, including VA health care

1, Covered | 2, Not covered | 3, Not sure

- Indian Health Service

1, Covered | 2, Not covered | 3, Not sure

- Any Other type of health insurance coverage or health coverage plan?

1, Covered | 2, Not covered | 3, Not sure

- Does this mean you currently have no health insurance or health coverage plan? In answering this question, please exclude plans that pay for only one type of service (such as, nursing home care, accidents, family planning, or dental care) and plans that only provide extra cash when hospitalized.

1, do not have health insurance | 2, have some kind of health insurance

- What type of health insurance do you have? (text)

LLM
\# Define a function to map the values from the project variable to the CDE variable

map_health_coverage <- function(value) {

  # Split the input string into a vector of character responses

  responses <- unlist(strsplit(as.character(value), ", "))

  # Check for employer or union insurance coverage

  if ("1" %in% responses) { # "1" indicates coverage through employer/union

    return(1) # 1 corresponds to "Covered"

  } else if ("8" %in% responses) { # "8" indicates none of the above

    return(2) # 2 corresponds to "Not Covered"

  } else if (any(c("77", "88") %in% responses)) { # "77" and "88" are for don't know/prefer not to answer

    return(3) # 3 corresponds to "Not Sure"

  } else {

    return(NA) # Return NA for missing or unrecognized responses

  }

}

(omitted)
